# Artificial Neural Network Modeling on PM_10_, PM_2.5_, and NO_2_ Concentrations between Two Megacities without a Lockdown in Korea, for the COVID-19 Pandemic Period of 2020

**DOI:** 10.3390/ijerph192316338

**Published:** 2022-12-06

**Authors:** Soo-Min Choi, Hyo Choi

**Affiliations:** 1 Department of Computer Engineering, Konkuk University, Chungju 27478, Republic of Korea; 2 Atmospheric and Oceanic Disaster Research Institute, Gangneung 25563, Republic of Korea

**Keywords:** artificial neural network model, COVID-19 pandemic, air quality, PM_10_, PM_2.5_, NO_2_, root mean square error, coefficient of determination

## Abstract

The mutual relationship among daily averaged PM_10_, PM_2.5_, and NO_2_ concentrations in two megacities (Seoul and Busan) connected by the busiest highway in Korea was investigated using an artificial neural network model (ANN)-sigmoid function, for a novel coronavirus (COVID-19) pandemic period from 1 January to 31 December 2020. Daily and weekly mean concentrations of NO_2_ in 2020 under neither locked down cities, nor limitation of the activities of vehicles and people by the Korean Government have decreased by about 15%, and 12% in Seoul, and Busan cities, than the ones in 2019, respectively. PM _10_ (PM_2.5_) concentration has also decreased by 15% (10%), and 12% (10%) in Seoul, and Busan, with a similar decline of NO_2_, causing an improvement in air quality in each city. Multilayer perception (MLP), which has a back-propagation training algorithm for a feed-forward artificial neural network technique with a sigmoid activation function was adopted to predict daily averaged PM_10_, PM_2.5_, and NO_2_ concentrations in two cities with their interplay. Root mean square error (RMSE) with the coefficient of determination (R^2^) evaluates the performance of the model between the predicted and measured values of daily mean PM_10_, PM_2.5_, and NO_2,_ in Seoul were 2.251 with 0.882 (1.909 with 0.896; 1.913 with 0.892), 0.717 with 0.925 (0.955 with 0.930; 0.955 with 0.922), and 3.502 with 0.729 (2.808 with 0.746; 3.481 with 0.734), in 2 (5; 7) nodes in a single hidden layer. Similarly, they in Busan were 2.155 with 0.853 (1.519 with 0.896; 1.649 with 0.869), 0.692 with 0.914 (0.891 with 0.910; 1.211 with 0.883), and 2.747 with 0.667 (2.277 with 0.669; 2.137 with 0.689), respectively. The closeness of the predicted values to the observed ones shows a very high Pearson r correlation coefficient of over 0.932, except for 0.818 of NO_2_ in Busan. Modeling performance using IBM SPSS-v27 software on daily averaged PM_10_, PM_2.5_, and NO_2_ concentrations in each city were compared by scatter plots and their daily distributions between predicted and observed values.

## 1. Introduction

Since the first patient of COVID-19 (Corona virus disease 2019) of unknown origin was first reported by the National Health Commission of the People’s Republic of China (NHC) on 31 December 2019 [1], and month after month, a dramatic increase in the number of COVID-19 patients was found, the Chinese government officially closed urban transportation system in Wuhan on 23 January 2020, and all 31 provincial regions in Chinese mainland began initiating their first-level response to a significant public health emergency [2]. Thereafter, WHO [3] indicated that the global pandemic of COVID has continued even until 2022, and COVID-19 has rapidly spread out all over the world with a total accumulative number of patients exceeding 90,000 in China to 7,000,000 in the world until July 2020. 

In China [4,5,6,7,8,9,10,11,12,13], India [14,15,16,17], European countries of Italy and Spain [18,19,20,21], and the USA [22], the lockdown introduced to stop the spread of COVID-19 could result in a remarkable impact such as a decrease of industrial production and the restrictions on social activities by no going out of the house, but resultantly, the strict implementation of this measure by the Chinese government could improve urban air quality state by reducing pollutant emissions from vehicles on the road and factories in the industrial regions. Similarly, the occurrence of improved urban air quality state was found during the 2008 Beijing Olympic Games [23,24], Asia-Pacific Economy Cooperation (APEC)- China Summit [25,26], and National holiday [27]. 

**Figure 1:**
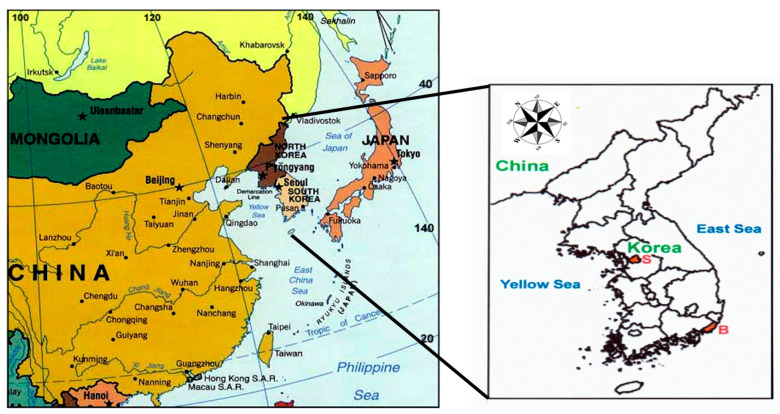
Location of two megacities, Seoul and Busan (colorful districts) on the left of S, and B which are connected with the busiest highway of about 430km in South Korea.

Even though most of the countries are locked down to prevent the spreading of this disease inside their countries, in contrast, only one country, South Korea has made an effort for overcoming the spread of this disease with no lockdown in any cities (Figure 1). After the first patient was found in February 2020 in Korea, the total accumulative number of patients infected from COVID-19 exceeded 415,000 with the death of 3274 persons and a weekly average of 2852 patients with a dramatic increase of COVID-19 patient number under the increase of virus variants until December 2021 [28,29,30]. However, this measure by the Korean government without any restriction on social activity, public and private transportation and even no entry ban for foreigners, which was different from the strict Chinese measure like a lockdown of the society have successfully controlled the spreading of COVID-19 epidemic inside Korea, no showing a dramatic increase of COVID patients, compared to any other advanced countries such as European countries having a similar population of Korea [31]. 

Korean government’s treatment to successfully control COVID-19 epidemic inside Korea is as follows. The Korean government has increased the vaccination rate of all citizens to more than 90% to prevent the spread of COVID-19. As South Korea has the largest number of hospitals relative to its population in the world, medical insurance is provided to all citizens 100%, free vaccination 4 times (every 3 months), free admission to the hospital by ambulance by the 119-patient transportation system in case of a patient, and treatment until the patient is completely cured [30].

Similar to the improvement of urban air quality in mega cities in China, European countries, and India during a COVID-19 pandemic period, it was found that the concentrations of major air pollutants such as PM_10_, PM_2.5_, and NO_2_ in megacities (Seoul and Busan) of South Korea decreased in 2020 rather than in 2019 with no COVID-19 pandemic, due to the people themselves limiting social activities and reducing the operation of automobiles, resulting in the improvement of air quality. For example, the reduction rates of NO_2_, PM_10_, and PM_2.5_ concentrations in Seoul (Busan) in South Korea were 15% (12%), 15% (10%), and 12% (10%), respectively, as shown later, while the rates of NO_2_, and PM_2.5_ in Wuhan, China were 53% and 35% [13], with their different reduction rates of pollution in different countries, respectively.

**Figure 2:**
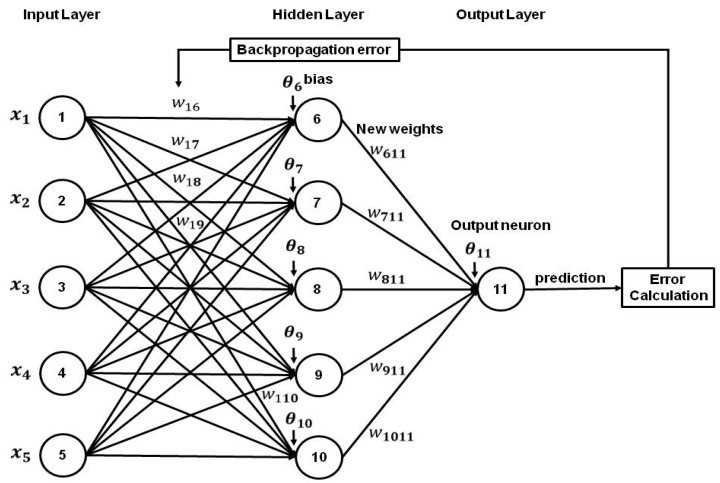
The structure of multilayer perceptron (MLP). Five sets of input data are initially fed into an input layer and the desired output is obtained from the output layer through the hidden layer. The number of neurons in the hidden layer is varied such as 2, 5, and 7 in our study, and the corresponding statistical indicators are recorded.

The objective of this study is to derive a practical formula for the prediction of air quality such as representative air pollutants of PM_10_, PM_2.5_, and NO_2_, using an artificial neural network model (ANN)-sigmoid activation function (Figure 2), and to know how much three pollutant concentrations in one city can affect the concentrations in another city between two megacities of Seoul and Busan for a COVID-19 pandemic period of 2020. The measured values of air pollutant variables were compared with the predicted values by the ANN model with different nodes in its hidden layer using SPSS-v27 software.

## 2. Materials and Methods

### 2.1. Study Location 

Seoul inside Gyeonggi province is the capital and largest metropolis of South Korea. Seoul has a population of 10.5 million people in the heart of the Seoul Capital Area. However, the population of a great Seoul is about 25 million people including the surrounding 8 satellite cities, and it covers about half of the total population of 52 million in South Korea (Figure 1). Seoul proper comprises 605.25 km^2^ with a radius of approximately 15 km, roughly bisected into northern and southern halves by the Han River. The latitude of Seoul is 37.533° N, and the longitude is 127.025° E.

Busan inside Kyungnam province officially known as Busan Metropolitan City is South Korea’s second-most populous city after Seoul, with a population of over 3.7 million inhabitants, but its population is about 8 million people including the people of the surrounding 3 satellite cities. Busan covers an area of 765.82 km^2^ and is the biggest port city in South Korea. It is an important business, sports, and cultural hub, as well as the second most crowded city after Seoul. Located on the shores of the Korea Strait, the city is one of the major ports and a key transport knot. Its latitude and longitude coordinates are 35.167° N, and 129.067° E. Seoul and Busan in South Korea are connected by the busiest highway of about 430km on which a huge number of vehicles move.

For the period of the COVID-19 pandemic of 2020, PM_10_, PM_2.5_, and NO_2_ concentrations in the two cities were much reduced compared with those concentrations of 2019, and those concentrations in the two cities showed similar patterns. A more detailed analysis of those pollutant concentrations, considering their mutual relation in the two cities in 2020 is given later. 

### 2.2. Air Quality Data Analysis

The characteristics in daily and weekly temporal variations and spatial distribution of NO_2_ and PM_10_, PM_2.5_ concentrations were further investigated in these cities. This study could have considerable implications for air pollution control in the megacities of Korea. More details of the specifications and procedures of the measurements for daily air quality data supplied by Korea Environment Corporation (KEC) are listed on the website. (https://www.airkorea.or.kr/web) (accessed on 10 January 2021) [32]. In this study, daily mean concentrations of nitrogen dioxide (NO_2_) and particulate matter with an aerodynamic diameter of fewer than 2.5 μm (PM_2.5_) particulate matter with an aerodynamic diameter of fewer than 10 μm (PM_10_) at Seoul and Busan cities were obtained from the KEC. The data of air quality have each unit as ppm × 1000 for NO_2_ concentration, μg/m^3^ for PM_10_ and PM_2.5_ concentrations.

In another way, each researcher can download air quality data without any restrictions after registering personal information through the National Institute of Environmental Research (NIER), the Ministry of Environment of the Korean Government with a website address as https://www.nier.go.kr/NIER/kor/openapi/ (accessed on 10 January 2021) [33]. Especially, all kinds of information on the COVID-19 epidemic to the public are supplied in real-time by the Korea Disease Control and Prevention Agency (KDCPA) (accessed on 10 January 2021) [30], such as the information on the nationwide supply and demand of the coronavirus vaccine, the date of individual vaccination, the number of infected patients and the number of deaths and others through a website, https://www.kdca.go.kr/index.es?sid=a3 (accessed on 10 January 2021). For this study, the daily number of patients infected by the COVD-19 epidemic in two megacities was obtained by https://www.seoul.go.kr/coronaV/coronaStatus.do (accessed on 10 January 2021) for Seoul, and https://www.busan.go.kr/covid19/Status01.do (access on 10 January 2021) for Busan.

### 2.3. Artificial Neural Network (ANN)Model—Machine Learning Model

A machine learning multilayer perceptron (MLP) with a forward artificial neural network is adopted among some of the ANN topologies available in the literature, in order to predict daily mean concentrations of particulate matter (PM_10_, PM_2.5_) and nitrogen dioxide (NO_2_) in two cities. The ANN model using the MLP is trained with the back-propagation training algorithm for feed-forward ANN [34], and it consists of an input layer, a hidden layer, and an output layer. Each layer processes the input data through the activation function. 

The ANNs contain numerical modeling techniques that attempt to simulate the behavior of the human brain and nervous system [35,36]. As a computational model, it consists of several processing elements that receive inputs and deliver outputs based on their predefined activation functions. 

A multilayer artificial neuron network is an integral part of deep learning. MLP is particularly useful in modeling to resolve a complex problem. Figure 2 illustrates the structure of MLP which consists of an input layer, a hidden layer, and an output layer. Input signals are multiplied by a set of weights as they are sent to the output layer through the hidden layer. For the calculation of the output of neurons from both the input layer to the hidden layer and the hidden layer to the output layer, a feed-forward artificial neural network with sigmoid activation function such as Equations (2) and (3) was adopted in two kinds of transferring process as a typical nonlinear function. 

The database used for the ANN model development comprises a total of 2196 hourly averaged data on 3 variables (PM_10_, PM_2.5_, and NO_2_) of two cities for 366 days. The ranges of data used for the input and output variables are summarized in Table 1. The available data were divided into three sets such as training, testing, and validation for their statistical consistency. 60%, 20%, and 20% of the total data in this study were randomly split for training, testing, and validation, similar to Choi [36], respectively. Before presenting the input and output variables for the ANN model training, they were scaled between 0.0 and 1.0 to eliminate their dimension, using Equation (1) and to ensure that all variables receive equal attention during training. 

We adopted the simple linear mapping of the variables’ practical extremes to the neural network’s practical extremes for data scaling suggested by Masters [37], Shahin, et al. [35], and Choi [36], because it is used as the most common method. For instance, in the case of each variable x with maximum and minimum values of $x_{max}$ and $x_{min}$, the scaled value $x_{n}$ is calculated by

(1) $$x_{n}=\frac{\left(x-x_{min}\right)}{\left(x_{max}-x_{min}\right)}$$

Similar to the way performed by Shahin et al. [35], and Choi [36], we determined an optimal model structure using a trial-and-error approach in which the ANN models were trained using one hidden layer with 2, 5, and 7 nodes, respectively.

The output of neuron *j* ($y_{j}$) in the typical MLP with a single hidden layer can be modeled by an activation function. Generally, the actuation function contains a multivariate linear function, an exponential function like a sigmoid, and a s-shaped function like a hyperbolic tangent. Among them, we adopted sigmoid as a typical nonlinear activation function for the calculation of the output of neurons by Equations (2) and (3), suggested by Choi [36], and the final output ($y_{11}$) of the neuron in the output layer can also be calculated by a sigmoid activation function. 

The output of neuron *j* is given by

(2) $$y_{j}=f\;\left(\theta_{j}+\sum_{j=6}^{p}w_{i,j}x_{i}\;\right)$$

(3) $$y_{j}=f\left(x\right)=sigmoid\left(x\right)=\frac{1}{\left(1+\exp\left(-x\right)\right)}$$

where f, *θ*, $x_{i}$, $y_{j}$ and w, are the activation function (or transfer function; here, sigmoid), bias (unit), input data, output of neuron, and weight coefficients. In the case of 5 neurons, the different weights are given not only form 5 input data to each neuron on 5 units in a single hidden layer, but also given from 5 units in the hidden layer to 1 unit in the output layer, which control the contribution of neuron, by carrying out back-propagation to adjust weights in a neural network. Here, *i (j)* is in the range of 1 to p (6 to p), which is 8 (11 and 13) in 2 (5 and 7) nodes in the hidden layer, and an output from one layer is an input into the next layer. 

In the case of five input data, the output of each neuron on five units in the hidden layer and one unit in the output layer can be written, as below. It means that using input data of $x_{1}$ to $x_{5}$, the outputs of neurons $x_{6}$ to $x_{10}$, such as $y_{6}$ to $y_{10}$ in the hidden layer are calculated by

(4) $$y_{6}=\frac{1}{1+\exp[-(\;\theta_{6}+W_{16}\;x_{1}+W_{26}\;x_{2}+W_{36}\;x_{3}+W_{46}\;x_{4}+W_{56}\;x_{5})]}$$

(5) $$y_{7}=\frac{1}{1+\exp[-(\;\theta_{7}+W_{17}\;x_{1}+W_{27}\;x_{2}+W_{37}\;x_{3}+W_{47}\;x_{4}+W_{57}\;x_{5})]}$$

(6) $$y_{8}=\frac{1}{1+\exp[-(\;\theta_{8}+W_{18}\;x_{1}+W_{28}\;x_{2}+W_{38}\;x_{3}+W_{48}\;x_{4}+W_{58}\;x_{5})]}$$

(7) $$y_{9}=\frac{1}{1+\exp[-(\;\theta_{9}+W_{19}\;x_{1}+W_{29}\;x_{2}+W_{39}\;x_{3}+W_{49}\;x_{4}+\;W_{59}\;x_{5})]}$$

(8) $$y_{10}=\frac{1}{1+\exp[-(\;\theta_{10}+\;W_{110}\;x_{1}+W_{210}\;x_{2}+W_{310}\;x_{3}+W_{410}\;x_{4}+W_{510}\;x_{5})]}$$

where input variables of $x_{1}$, $x_{2}$, $x_{3}$, $x_{4}$, and $x_{5}$ denote the concentrations of PM_2.5_ and NO_2_ at Seoul city, and PM_10_, PM_2.5_, and NO_2_ at Busan city. The outputs of neurons $x_{11}$, that is, $y_{11}$ as PM_10_ at Seoul city in the output layer, using output values ($y_{6}$ to $y_{10}$) of $x_{6}$ to $x_{10}$ in the hidden layer are calculated by

(9) $$y_{11}=\frac{1}{1+\exp[-(\;\theta_{11}+W_{611}\;y_{\;6}+W_{711}\;y_{7}+W_{811}\;y_{8}+W_{911}\;y_{9}+W_{1011}\;y_{10})]}$$

In the training architecture of the ANN model, the initial values of lambda and sigma are 0.0000005 and 0.00005. 

Previously, before using Equations (2) and (3), all input variables were scaled between 0.0 to 1.0 using Equation (1) in the range between the maximum and minimum values of the input data. As the calculated values from Equation (9) were scaled between 0.0 and 1.0, these calculated values should be re-scaled again for obtaining the actual values, using Equation (1), after the calculation of output in the output layer. In the case of seven units (nodes) in the hidden layer and one unit in the output layer can be written slightly differently from the case of five nodes in the hidden layer like $y_{13}$.

## 3. Results

### 3.1. Temporal Variations of PM_10_, PM_2.5_, and NO_2_ Concentrations at Two Cities 

**Figure 3:**
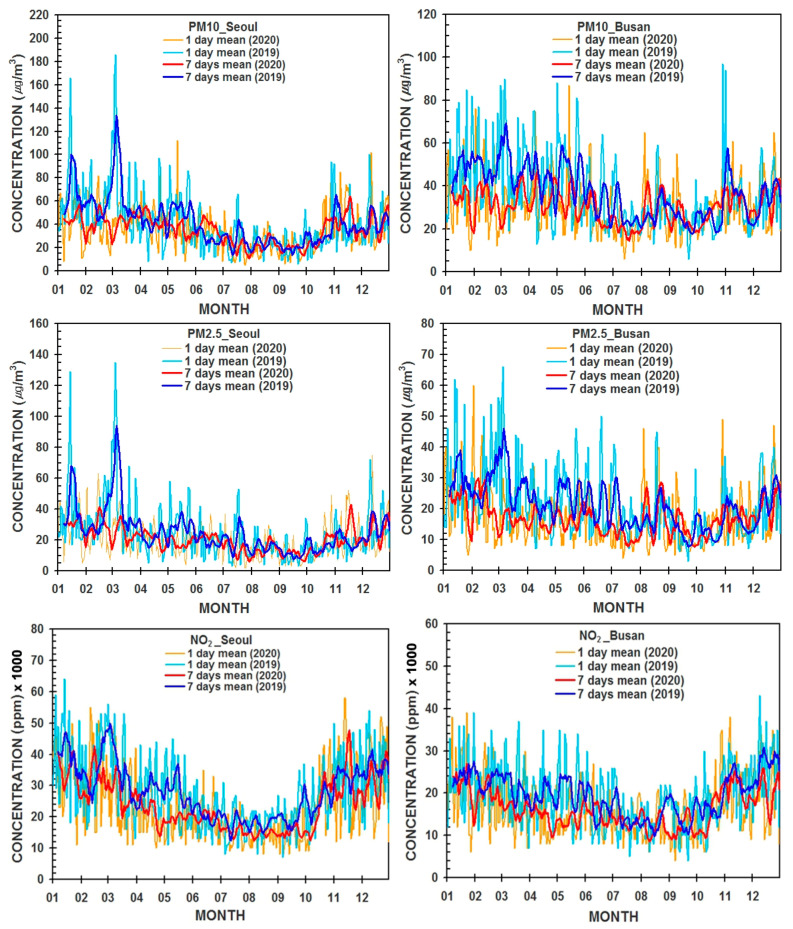
Temporal variations of measured daily and seven days averaged concentrations of PM_10_ (μg/m^3^), PM_2.5_ (μg/m^3^), and NO_2_ (ppm ×1000) before (2019) and during the COVID-19 epidemic year (2020) in Seoul and Busan cities, Korea.

Figure 3 shows the temporal variations of daily and weekly mean values of PM_10_, PM_2.5_, and NO_2_ concentrations from 1 January to 31 December 31 2019 (one year before the COVID-19 pandemic) and 2020 (during the COVID-19 pandemic) in Seoul (the capital city) and Busan (the second largest city) in Korea. In order to give the importance of the NO_2_ concentration similar to the PM_10_ and PM_2.5_ concentrations, the NO_2_ concentration was used by multiplying it by 1000 in our ANN models.

Even though the variation tendency of the daily average of PM_10_, PM_2.5_, and NO_2_ concentration in Busan city is similar to that in Seoul city, their magnitudes in Busan were much smaller than those in Seoul city. It may be attributed to the different populations of two cities such as about 7 million (Busan) with its suburban cities and 25 million (Seoul) with surrounding satellite cities. 

Weekly mean values of PM_10_, PM_2.5_, and NO_2_ concentrations in Seoul have similar tendencies to their daily mean values in 2019 and 2020 in Seoul and Busan. Daily and weekly mean concentrations of NO_2_ in 2020 have slightly decreased by about 15% (Seoul), and 12% (Busan), compared to ones in 2019, respectively. PM_10_ (PM_2.5_) concentration has also decreased by 15% (10%) in Seoul, and 12% (10%) in Busan, showing a similar significant decline of NO_2_. These reduction patterns of PM_2.5_ and NO_2_ concentration in two cities with much lower concentrations were similar to Wuhan, Hubei (Wuhan excluded), and China (Hubei excluded), where the first infected patient was found [38]. 

It was known that a concurrent significant change in air pollutant concentrations in China was with the introduction of control measures and societal lockdowns to limit COVID-19 spread. Chu, et al. [13], and Wang, et al. [39] insisted that especially, the reduction of NO_2_ was attributed to the significant reductions (40~80%) of city traffic in eastern and northern China during the COVID-19 epidemic. 

Different from the Chinese government, the Korean government neither locked down cities, nor the limitation of the activities of vehicles and people, but individuals overall restrained their activities, resulting in a slight decrease of over 10% in air pollutant concentrations in 2020, and the improvement of air quality in each city. 

### 3.2. Evaluation of Daily Mean PM_10_ and PM_2.5_, and NO_2_ Concentrations Using an ANN Model 

For model training, testing, and validation, daily mean data of PM_10_, PM_2.5_, and NO_2_ concentrations for one year was divided into two datasets. Initial 60% of the data was utilized for the development of ML models as training, 20% for testing the model, and the rest 20% was used for the model assessment (validation), similar to the performance by Shahin, et al. [35], Nawras and Hani [40], and Choi [36]. 

For the calculation of PM_10_ (Seoul), not only PM_2.5_ (Seoul), and NO_2_ (Seoul) in Seoul city, but also PM_10_ (Busan), PM_2.5_ (Busan), and NO_2_ (Busan) in Busan city were used as input data for the development of the MLP model. In order to calculate another variable, the remaining variables were used as input data and performed in the same way sequentially. 

Table 1, Table 2 and Table 3 indicate the weights and threshold values for different nodes such as 2, 5, and 7 in a single hidden layer for the prediction of PM_10_, PM_2.5_, and NO_2_ concentrations transferred by a sigmoid activation function of the ANN model from the input layer to the hidden layer and from the hidden layer to the output layer, respectively, in detail. For instance, the output PM_10_ ($y_{11}$) at Seoul in a case of 5 nodes of the hidden layer can be calculated using output values of $y_{6}$ to $y_{10},$ as below. 

The performance of the neural network is analyzed by differing the number of neurons in the hidden layer such as 2, 5, and 7, here, and recording the respective statistical indicators. Neural Network application in SPSS-version 27 was adopted to design, train and validate the neural network model. As the most well-known feed-forward network, the MLP of which the back-propagation algorithm for error calculation is utilized to train the network is adopted in the current study of the ANN model. The model training terminates, when the generalization demonstrated by an increase in the mean square error (MSE) and the corresponding decrease in R^2^ stops its improvement. 

Namely, the loss function (or coast function) expressed by the mean square error formula (MSE) as shown in Equation (17) is used to calculate the error between the output value (predicted value) and the measured value (true value) in the back-propagation algorithm. If the predicted value is not close to the measured value, it goes back to the ANN model circuit and adjusts the weight again. Then it goes through the hidden layer again to the output layer, calculating the output value in the output layer. If the output value is very close to the measured value, the calculation of the output value is terminated.

**Table 1:** Parameter estimates of weights and threshold values in the case of 2 nodes in one hidden layer in Seoul and Busan cities.

**Parameter Estimates-PM_10_(Seoul)-Node=2**						**Parameter Estimates-PM_10_(Busan)-Node=2**				
		**Hidden Layer 1**		**Output** **Layer**				**Hidden Layer 1**		**Output** **Layer**
**Predictor**		**H(1:1)**	**H(1:2)**	**PM_10__S**		**Predictor**		**H(1:1)**	**H(1:2)**	**PM_10__B**
**Input** **Layer**	**(Bias)**	−0.297	−0.051			**Input** **Layer**	**(Bias)**	0.458	0.230	
	**PM_2.5__S**	1.201	−0.871				**PM_2.5__B**	−1.278	−1.130	
	**NO_2__S**	−0.334	−0.187				**NO_2__B**	0.076	0.206	
	**PM_10__B**	0.337	−1.408				**PM_10__S**	−1.154	−0.949	
	**PM_2.5__B**	−0.669	0.915				**PM_2.5__S**	1.423	0.512	
	**NO_2__B**	0.196	−0.059				**NO_2__S**	−0.063	−0.145	
**Hidden** **Layer 1**	**(Bias)**			0.189		**Hidden** **Layer 1**	**(Bias)**			2.200
	**H(1:1)**			2.262			**H(1:1)**			−2.381
	**H(1:2)**			−2.269			**H(1:2)**			−1.400
										
**Parameter Estimates-PM_2.5_(Seoul)-Node=2**						**Parameter Estimates-PM_2.5_(** **Busan)-Node=2**				
		**Hidden Layer 1**		**Output** **Layer**				**Hidden Layer 1**		**Output** **Layer**
**Predictor**		**H(1:1)**	**H(1:2)**	**PM_2.5__S**		**Predictor**		**H(1:1)**	**H(1:2)**	**PM_2.5__B**
**Input** **Layer**	**(Bias)**	0.194	−1.107			**Input** **Layer**	**(Bias)**	−1.298	0.002	
	**PM_10__S**	−1.778	−0.262				**PM_10__B**	0.906	3.675	
	**NO_2__S**	0.427	0.947				**NO_2__B**	0.161	2.252	
	**PM_10__B**	1.142	0.298				**PM_10__S**	−1.083	1.542	
	**PM_2.5__B**	−0.659	0.652				**PM_2.5__S**	0.986	2.774	
	**NO_2__B**	0.122	−0.037				**NO_2__S**	−0.089	1.139	
**Hidden** **Layer 1**	**(Bias)**			0.925		**Hidden** **Layer 1**	**(Bias)**			−1.389
	**H(1:1)**			−2.784			**H(1:1)**			4.984
	**H(1:2)**			2.062			**H(1:2)**			0.386
										
**Parameter Estimates-NO_2_(Seoul)-Node=2**						**Parameter Estimates-NO_2_(** **Busan)-Node=2**				
		**Hidden Layer 1**		**Output** **Layer**				**Hidden Layer 1**		**Output** **Layer**
**Predictor**		**H(1:1)**	**H(1:2)**	**NO_2__S**		**Predictor**		**H(1:1)**	**H(1:2)**	**NO_2__B**
**Input** **Layer**	**(Bias)**	0.194	−0.754			**Input** **Layer**	**(Bias)**	0.026	1.537	
	**PM_10__S**	−0.850	0.039				**PM_10__B**	0.056	−0.118	
	**PM_2.5__S**	−0.492	0.725				**PM_2.5__B**	−0.840	−1.292	
	**PM_10__B**	−0.047	−0.388				**PM_10__S**	0.116	0.035	
	**PM_2.5__B**	−0.082	−0.222				**PM_2.5__S**	0.162	0.636	
	**NO_2__B**	−1.174	1.142				**NO_2__S**	−2.019	−1.376	
**Hidden** **Layer 1**	**(Bias)**			−0.538		**Hidden** **Layer 1**	**(Bias)**			1.670
	**H(1:1)**			−0.635			**H(1:1)**			−0.871
	**H(1:2)**			2.501			**H(1:2)**			−1.657

**Table 2:** As shown in Table 1, except for 5 nodes in a single hidden layer in Seoul and Busan cities.

**Parameter Estimates-PM_10_(Seoul)-Node=5**									**Parameter Estimates-PM_10_(Busan)-Node=5**							
**Hidden Layer 1**							**Output** **Layer**		**Hidden Layer 1**							**Output** **Layer**
**Predictor**		**H(1:1)**	**H(1:2)**	**H(1:3)**	**H(1:4)**	**H(1:5)**	**PM_10__S**		**Predictor**		**H(1:1)**	**H(1:2)**	**H(1:3)**	**H(1:4)**	**H(1:5)**	**PM_10__B**
**Input** **Layer**	**(Bias)**	0.498	0.155	−0.241	0.029	−0.408			**Input** **Layer**	**(Bias)**	−0.370	−0.670	0.032	0.157	−0.132	
	**PM_2.5__S**	−0.804	−1.172	−0.598	1.131	0.649				**PM_2.5__B**	−0.895	0.827	0.681	−0.812	0.937	
	**NO_2__S**	0.198	0.070	−0.097	−0.024	−0.031				**NO_2__B**	−0.023	−0.198	−0.008	−0.197	0.086	
	**PM_10__B**	−0.133	−0.882	−0.237	1.081	0.424				**PM_10__S**	0.273	1.460	0.448	−0.379	−0.436	
	**PM_2.5__B**	0.107	0.700	0.352	−1.068	−0.356				**PM_2.5__S**	0.061	−1.192	0.788	−0.401	−0.044	
	**NO_2__B**	0.086	0.318	−0.392	0.246	0.135				**NO_2__S**	0.026	0.212	0.441	0.099	−0.406	
**Hidden** **Layer 1**	**(Bias)**						0.134		**Hidden** **Layer 1**	**(Bias)**						−1.184
	**H(1:1)**						−0.485			**H(1:1)**						−0.766
	**H(1:2)**						−1.284			**H(1:2)**						3.524
	**H(1:3)**						−0.522			**H(1:3)**						−0.108
	**H(1:4)**						1.585			**H(1:4)**						−0.296
	**H(1:5)**						0.789			**H(1:5)**						1.009
																
**Parameter Estimates-PM_2.5_(Seoul)-Node=5**									**Parameter Estimates-PM_2.5_(Busan)-Node=5**							
**Hidden Layer 1**							**Output** **Layer**		**Hidden Layer 1**							**Output** **Layer**
**Predictor**		**H(1:1)**	**H(1:2)**	**H(1:3)**	**H(1:4)**	**H(1:5)**	**PM_2.5__S**		**Predictor**		**H(1:1)**	**H(1:2)**	**H(1:3)**	**H(1:4)**	**H(1:5)**	**PM_2.5__B**
**Input** **Layer**	**(Bias)**	−0.075	−0.861	1.332	−0.173	−0.923			**Input** **Layer**	**(Bias)**	0.145	−0.104	−0.383	−1.881	−0.312	
	**PM_10__S**	−0.312	0.518	−0.238	1.866	1.008				**PM_10__B**	1.510	0.240	−0.865	0.742	−0.021	
	**NO_2__S**	1.090	0.103	−0.708	−1.181	0.012				**NO_2__B**	0.220	0.246	−0.199	0.288	−0.019	
	**PM_10__B**	0.140	−1.284	0.430	−0.397	−0.576				**PM_10__S**	0.095	−0.237	0.360	−1.573	0.261	
	**PM_2.5__B**	0.607	0.880	−0.729	0.009	0.511				**PM_2.5__S**	−0.176	0.857	−0.228	1.482	0.279	
	**NO_2__B**	−0.039	0.156	0.398	0.032	−0.136				**NO_2__S**	−0.040	0.120	−0.244	−0.527	−0.268	
**Hidden** **Layer 1**	**(Bias)**						−0.892		**Hidden** **Layer 1**	**(Bias)**						−1.361
	**H(1:1)**						1.202			**H(1:1)**						1.175
	**H(1:2)**						1.277			**H(1:2)**						1.066
	**H(1:3)**						−1.468			**H(1:3)**						−0.756
	**H(1:4)**						1.652			**H(1:4)**						3.134
	**H(1:5)**						0.936			**H(1:5)**						0.256
																
**Parameter Estimates-NO_2_(Seoul)-Node=5**									**Parameter Estimates-NO_2_(Busan)-Node=5**							
**Hidden Layer 1**							**Output** **Layer**		**Hidden Layer 1**							**Output** **Layer**
**Predictor**		**H(1:1)**	**H(1:2)**	**H(1:3)**	**H(1:4)**	**H(1:5)**	**NO_2__S**		**Predictor**		**H(1:1)**	**H(1:2)**	**H(1:3)**	**H(1:4)**	**H(1:5)**	**NO_2__B**
**Input** **Layer**	**(Bias)**	0.134	−0.003	−0.774	−0.273	0.069			**Input** **Layer**	**(Bias)**	−0.186	−0.031	0.003	−0.432	−0.181	
	**PM_10__S**	−0.392	−0.450	−0.133	−0.536	−0.224				**PM_10__B**	0.281	−0.002	−0.158	0.032	0.274	
	**PM_2.5__S**	−0.307	0.465	0.804	0.043	−0.484				**PM_2.5__B**	−0.660	0.574	0.410	0.751	−0.366	
	**PM_10__B**	0.152	0.234	0.207	0.493	−0.281				**PM_10__S**	−0.531	0.080	0.069	−0.088	0.518	
	**PM_2.5__B**	0.347	−0.574	−0.377	−0.242	0.508				**PM_2.5__S**	−0.668	0.464	−0.084	−0.478	0.011	
	**NO_2__B**	−0.743	0.957	0.859	−0.502	−0.314				**NO_2__S**	−0.317	1.006	0.797	1.010	0.231	
**Hidden** **Layer 1**	**(Bias)**						−0.075		**Hidden** **Layer 1**	**(Bias)**						−1.357
	**H(1:1)**						−0.973			**H(1:1)**						−0.199
	**H(1:2)**						1.219			**H(1:2)**						0.590
	**H(1:3)**						1.319			**H(1:3)**						0.872
	**H(1:4)**						−0.406			**H(1:4)**						1.816
	**H(1:5)**						−0.558			**H(1:5)**						0.091

**Table 3:** As shown in Table 1, except for 7 nodes in a single hidden layer in Seoul and Busan cities.

		**Parameter Estimates-PM_10_(Seoul)-Node=7**							**Output** **Layer**				**Parameter Estimates-PM_10_(Busan)-Node=7**							**Output** **Layer**
		**Hidden Layer 1**											**Hidden Layer 1**							
**Predictor**		**H(1:1)**	**H(1:2)**	**H(1:3)**	**H(1:4)**	**H(1:5)**	**H(1:6)**	**H(1:7)**	**PM_10__S**		**Predictor**		**H(1:1)**	**H(1:2)**	**H(1:3)**	**H(1:4)**	**H(1:5)**	**H(1:6)**	**H(1:7)**	**PM_10__B**
**Input** **Layer**	**(Bias)**	0.477	0.337	−0.379	−0.192	−0.023	−0.369	0.068			**Input** **Layer**	**(Bias)**	1.069	−0.770	−0.648	0.176	0.307	−0.285	−1.063	
	**PM_2.5__S**	−0.046	0.445	−0.709	−0.958	1.011	0.947	−1.066				**PM_2.5__B**	−0.972	0.561	0.957	−0.128	0.522	−0.550	0.861	
	**NO_2__S**	−0.264	−0.220	0.151	−0.328	−0.277	0.075	−0.155				**NO_2__B**	0.023	−0.111	0.020	−0.115	−0.241	−0.009	−0.008	
	**PM_10__B**	−0.422	0.248	−0.184	−0.387	0.122	0.880	−0.155				**PM_10__S**	−0.370	1.310	0.198	−0.987	−0.167	−0.603	0.964	
	**PM_2.5__B**	−0.085	−0.341	0.323	0.263	−0.149	−0.751	−0.087				**PM_2.5__S**	1.345	−0.739	−0.304	0.105	−0.296	0.343	−0.919	
	**NO_2__B**	0.444	−0.347	−0.050	−0.125	0.167	−0.102	−0.219				**NO_2__S**	−0.204	−0.276	0.152	−0.029	0.344	0.549	0.219	
**Hidden** **Layer 1**	**(Bias)**								0.200		**Hidden** **Layer 1**	**(Bias)**								−0.158
	**H(1:1)**								−0.355			**H(1:1)**								−1.248
	**H(1:2)**								0.561			**H(1:2)**								1.351
	**H(1:3)**								−0.710			**H(1:3)**								0.800
	**H(1:4)**								−0.909			**H(1:4)**								−0.460
	**H(1:5)**								0.625			**H(1:5)**								0.606
	**H(1:6)**								1.277			**H(1:6)**								−0.433
	**H(1:7)**								−0.798			**H(1:7)**								1.379
																				
		**Parameter Estimates-PM_2.5_(Seoul)-Node=7**							**Output** **Layer**				**Parameter Estimates-PM_2.5_(Busan)-Node=7**							**Output** **Layer**
		**Hidden Layer 1**											**Hidden Layer 1**							
**Predictor**		**H(1:1)**	**H(1:2)**	**H(1:3)**	**H(1:4)**	**H(1:5)**	**H(1:6)**	**H(1:7)**	**PM_2.5__S**		**Predictor**		**H(1:1)**	**H(1:2)**	**H(1:3)**	**H(1:4)**	**H(1:5)**	**H(1:6)**	**H(1:7)**	**PM_2.5__B**
**Input** **Layer**	**(Bias)**	0.363	−0.119	−0.245	0.004	−0.176	−0.594	−0.213			**Input** **Layer**	**(Bias)**	−0.212	0.242	−0.543	−0.006	−0.447	−0.372	0.151	
	**PM_10__S**	−0.919	0.202	−0.149	−1.127	−0.030	0.578	0.202				**PM_10__B**	0.147	−1.131	1.127	−0.403	0.133	0.141	0.584	
	**NO_2__S**	−0.020	0.175	−0.341	0.245	−0.087	0.495	0.084				**NO_2__B**	0.347	−0.176	0.275	−0.072	0.207	0.652	0.283	
	**PM_10__B**	0.139	−0.450	0.397	0.272	−1.009	−0.614	−0.644				**PM_10__S**	−0.032	0.657	−1.120	0.207	−0.099	0.135	−0.763	
	**PM_2.5__B**	−0.637	0.265	−0.440	−0.235	0.005	0.800	0.627				**PM_2.5__S**	−0.706	−0.358	1.134	−0.551	0.479	0.163	0.418	
	**NO_2__B**	0.170	0.101	−0.290	0.052	−0.710	−0.276	0.412				**NO_2__S**	−0.005	−0.116	−0.717	−0.225	−0.110	−0.115	0.423	
**Hidden** **Layer 1**	**(Bias)**								0.636		**Hidden** **Layer 1**	**(Bias)**								−0.299
	**H(1:1)**								−1.669			**H(1:1)**								−0.587
	**H(1:2)**								0.361			**H(1:2)**								−1.098
	**H(1:3)**								−0.812			**H(1:3)**								1.861
	**H(1:4)**								−1.468			**H(1:4)**								−0.545
	**H(1:5)**								0.876			**H(1:5)**								0.448
	**H(1:6)**								1.242			**H(1:6)**								0.242
	**H(1:7)**								0.859			**H(1:7)**								0.892
																				
		**Parameter Estimates-NO_2_(Seoul)-Node=7**							**Output** **Layer**				**Parameter Estimates-NO_2_(Busan)-Node=7**							**Output** **Layer**
		**Hidden Layer 1**											**Hidden Layer 1**							
**Predictor**		**H(1:1)**	**H(1:2)**	**H(1:3)**	**H(1:4)**	**H(1:5)**	**H(1:6)**	**H(1:7)**	**NO_2__S**		**Predictor**		**H(1:1)**	**H(1:2)**	**H(1:3)**	**H(1:4)**	**H(1:5)**	**H(1:6)**	**H(1:7)**	**NO_2__B**
**Input** **Layer**	**(Bias)**	−0.442	0.873	−0.430	0.055	−0.550	0.693	0.240			**Input** **Layer**	**(Bias)**	−0.265	−0.560	−0.074	0.053	−0.428	0.474	−0.474	
	**PM_10__S**	0.083	−0.136	−0.650	0.167	0.652	0.205	−0.238				**PM_10__B**	−0.024	−0.357	0.178	0.927	−0.392	−0.156	0.044	
	**PM_2.5__S**	0.287	−1.147	0.671	−0.039	0.391	−0.393	0.371				**PM_2.5__B**	−0.419	0.588	0.305	0.454	−0.533	0.450	0.464	
	**PM_10__B**	−0.234	0.469	0.448	−0.118	0.161	−0.231	0.085				**PM_10__S**	−0.152	−0.148	−0.156	0.793	−0.731	0.053	0.453	
	**PM_2.5__B**	0.114	0.233	0.221	−0.090	−0.638	0.341	−0.864				**PM_2.5__S**	−0.729	−0.389	0.337	0.718	−0.859	−0.049	−0.604	
	**NO_2__B**	−0.180	−1.042	0.321	−0.272	0.851	−0.629	0.728				**NO_2__S**	−0.943	0.647	−0.037	1.443	−1.103	0.433	1.082	
**Hidden** **Layer 1**	**(Bias)**								0.164		**Hidden** **Layer 1**	**(Bias)**								−0.197
	**H(1:1)**								−0.045			**H(1:1)**								−0.497
	**H(1:2)**								−1.189			**H(1:2)**								1.292
	**H(1:3)**								0.638			**H(1:3)**								−0.207
	**H(1:4)**								−0.130			**H(1:4)**								−1.121
	**H(1:5)**								0.921			**H(1:5)**								−1.084
	**H(1:6)**								−0.697			**H(1:6)**								0.249
	**H(1:7)**								1.009			**H(1:7)**								2.409

In the case of 5 nodes in the hidden layer, input variables of $x_{1}$, $x_{2}$, $x_{3}$, $x_{4}$, and $x_{5}$ denote the concentrations of PM_2.5_ and NO_2_ at Seoul, and PM_10_, PM_2.5_, and NO_2_ at Busan. The outputs of neurons $x_{11}$, namely, $y_{11}$ as PM_10_ at Seoul city in the output layer, using output values ($y_{6}$ to $y_{10}$) of $x_{6}$ to $x_{10}$ in the hidden layer are calculated as below.

(10) $$y_{6}=\frac{1}{1+\exp\;[-(\;0.498-0.804\;x_{1}+0.198\;x_{2}-0.133x_{3}+0.107x_{4}+0.086x_{5})]}$$

(11) $$y_{7}=\frac{1}{1+\exp\;[-(\;0.155-1.172x_{1}+0.07x_{2}-0.882x_{3}+0.70x_{4}+0.318x_{5})]}\;$$

(12) $$y_{8}=\frac{1}{1+\exp\;[-(-0.241-0.598x_{1}-0.097x_{2}-0.237x_{3}+0.352x_{4}-0.392x_{5})]}$$

(13) $$y_{9}=\frac{1}{1+\exp\;[-(0.029+1.131x_{1}-0.024x_{2}+1.081x_{3}-1.068x_{4}+0.246x_{5})]}$$

(14) $$y_{10}=\frac{1}{1+\exp\;[-(-0.408+0.649x_{1}-0.031x_{2}+0.424x_{3}-0.356x_{4}+0.135x_{5})]}$$

(15) $$y_{11}=\frac{1}{1+\exp\;[-(\;0.134-0.485y_{6}-1.284y_{7}-0.522y_{8}+1.585y_{9}+0.789y_{10})]}$$

In a similar way, the outputs of neurons, $y_{11}$ as PM_2.5_ and NO_2_ at Seoul city and PM_10_, PM_2.5,_ and NO_2_ at Busan city in the output layer, using different coefficients of output values ($y_{6}$ to $y_{10}$) of $x_{6}$ to $x_{10}$ in the hidden layer of Table 2 be calculated. 

In the case of 7 nodes in the hidden layer, input variables of $x_{1}$, $x_{2}$, $x_{3}$, $x_{4}$, and $x_{5}$ denote the concentrations of PM_2.5_ and NO_2_ at Seoul city, and PM_10_, PM_2.5_, and NO_2_ at Busan city. The outputs of neurons $x_{13}$, namely, $y_{13}$ as PM_10_ at Seoul city in the output layer, using output values ($y_{6}$ to $y_{12}$) of $x_{6}$ to $x_{12}$ in the hidden layer are calculated.

### 3.3. Statistical Performance of the Optimal ANN Model with Data Sets of Training, Testing, and Validation 

As the predicted values of each variable calculated from Equation (11) were scaled between 0.0 and 1.0, using Equation (1) before adopting machine learning techniques, these values should be re-scaled again for obtaining the actual predicted values, using Equation (1), after the calculation of output in the output layer. Then, these actual predicted values were used for the comparison of measured values.

**Figure 4:**
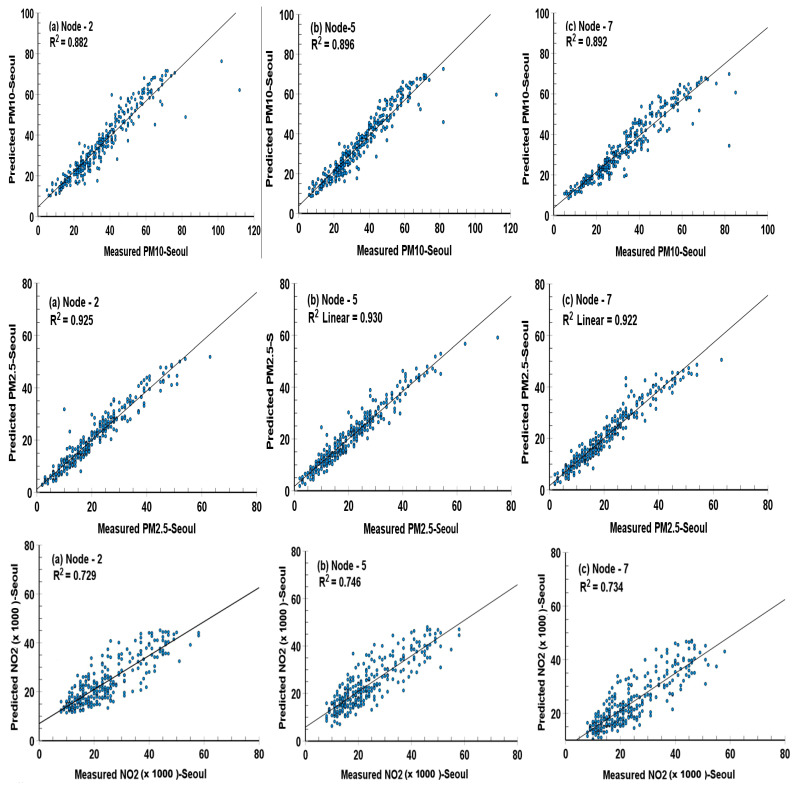
Scatter plots with correlations between measured and predicted daily mean concentrations of PM_10_ (μg/m^3^), PM_2.5_ (μg/m^3^), and NO_2_ (ppm × 1000), using different neuron number in the hidden layer-2, 5, and 7 in an ANN, based sigmoid activation function in Seoul city, Korea during the COVID-19 epidemic year (2020).

**Figure 5:**
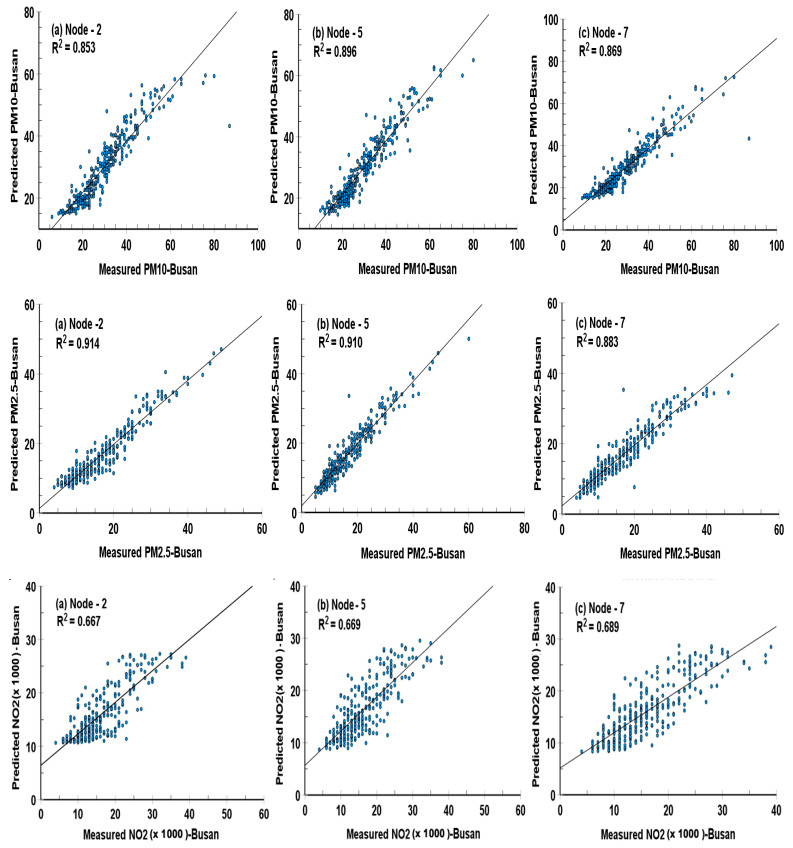
As shown in Figure 4, except for Busan city, Korea.

**Figure 6:**
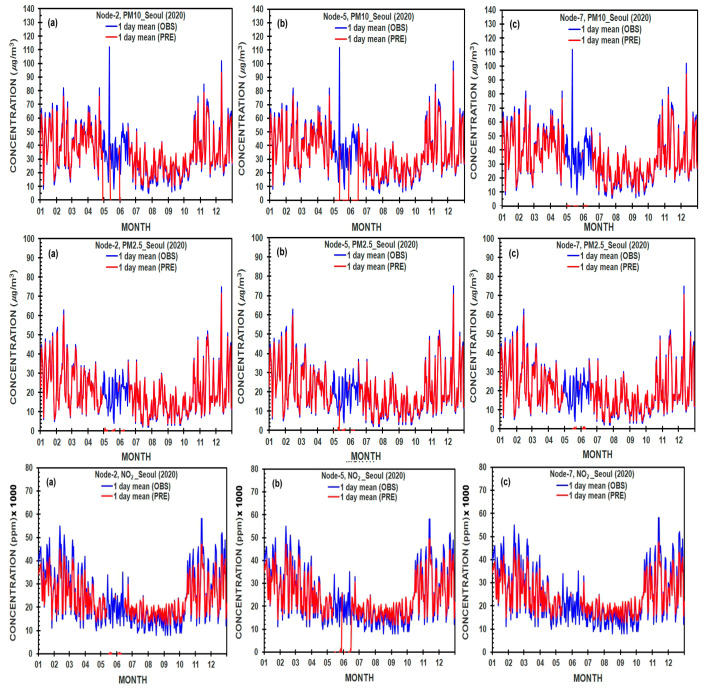
Comparison between predicted and measured daily averaged PM_10_ (μg/m^3^), PM_2.5_ (μg/m^3^), and NO_2_ (ppm × 1000), concentrations, using different numbers of hidden neurons-2, 5, and 7 in an Artificial Neural Network Model-based a sigmoid activation function at Seoul city, Korea, during the COVID-19 epidemic year (2020).

**Figure 7:**
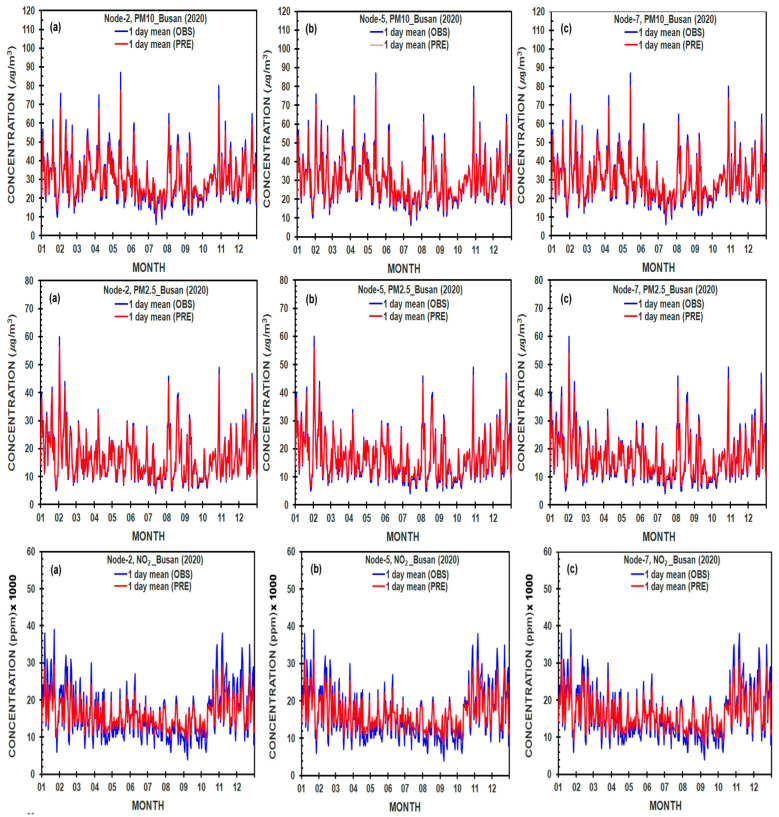
As shown in Figure 6, except for Busan city, Korea.

The result of the comparison of predicted and measured values of each variable in the different nodes in the hidden layer was given in Figure 4, Figure 5, Figure 6 and Figure 7 and Table 4 with root mean square error (RMSE), mean square error (MSE), and coefficient of determination (*R*^2^), which are utilized to evaluate the values of various variables by the following equations suggested by Dhakal, et al. [41]

(16) $$RMSE=\sqrt{\frac{1}{m}\sum_{i=1}^{m}{\left(Y_{i}-X_{i}\right)}^{2}}$$

(17) $$MSE=\frac{1}{m}\sum_{i=1}^{m}{\left(Y_{i}-X_{i}\right)}^{2}$$

(18) $$R^{2}=\frac{{\left[\sum_{i=1}^{m}\left(X_{i}-\bar{X}\right)\left(Y_{i}-\bar{Y}\right)\right]}^{2}}{\sum_{i=1}^{m}{\left(X_{i}-\bar{X}\right)}^{2}\sum_{i=1}^{m}{\left(Y_{i}-\bar{Y}\right)}^{2}}$$

where *X_i_*, *Y_i_*, and *m* represent the measured and predicted values, and the number of data, while $\bar{X}$ and $\bar{Y}$ represent the average measured and average estimated values. 

Table 4 provides a summary of the statistical indicators for ANN models adopting a sigmoid activation function from input to hidden layers. It shows the prediction performance of the optimal ANN model in the cases of 2, 5, and 7 nodes in a single hidden layer at two cities, and the validation of the predicted values to the measured values of PM_10_, PM_2.5_, and NO_2_ concentrations was presented by the statistical indicators such as RMSE and R^2^. 

For all data sets (training, testing, and validation), the models developed by us could perform well in the prediction of dependent variables of PM_10_, PM_2.5_, and NO_2_ at Seoul and Busan cities, even though NO_2_ concentration in Busan city had slightly lower R^2^ than other concentrations. Among them, the ANN model with 5 hidden nodes was the best to make the predicted values closest to the measured values, and with 7 hidden nodes was next followed. 

Focusing validation of the ANN model, coefficients of determination (R^2^; Pearson r^2^) between the measured and the calculated values of daily mean PM_10_, PM_2.5_, and NO_2_ at Seoul were 0.882 (0.896 and 0.892), 0.925 (0.930 and 0.922) and 0.729 (0.746 and 0.734), in 2 (5 and 7) nodes in a single hidden layer. The coefficients of PM_10_, PM_2.5_ and NO_2_ at Busan were 0.853 (0.896 and 0.869), 0.914 (0.910 and 0.883) and 0.667 (0.669 and 0.689), respectively. The closeness of the prediction to the observation that is given by R^2^ value shows greater than 93.2% in PM_10_ and PM_2.5_, and 81.7% in NO_2_ in the two cities. 

Prediction performance of the optimal ANN model in the different node numbers of 2, 5, and 7 in s single hidden layer at two cities was presented by RMSE and R^2^. Generally, a lower RMSE value corresponds to better performance for the prediction. R^2^ indicates the correlation coefficient of the dependent variables associated with an independent variable, and its higher value shows better prediction. Thus, as higher R^2^ generally well correspond to lower RMSE for all data sets (training, testing, and validation), the model developed by us performed well in the prediction of dependent variables, and ANN with 5 nodes was the best model in our study. 

**Table 4:** Performance validation of the optimal ANN model with 2, 5, and 7 hidden layer nodes. showing RMSE and R^2^.

Variables	Hidden Neuron Numbers	RMSE			R^2^		
		Training	Testing	Validation	Training	Testing	Validation
PM_10_-Seoul	2	2.438	2.251	2.251	0.864	0.894	0.882
	5	2.959	1.745	1.909	0.868	0.870	0.896
	7	1.904	1.738	1.913	0.892	0.884	0.892
PM_2.5_-Seoul	2	1.076	1.511	0.717	0.921	0.900	0.925
	5	0.836	1.193	0.955	0.932	0.904	0.930
	7	0.847	1.196	0.955	0.938	0.905	0.922
NO_2_-Seoul	2	3.369	3.305	3.502	0.704	0.699	0.729
	5	3.256	4.024	2808	0.720	0.685	0.746
	7	3.032	3.456	3.481	0.722	0.722	0.734
PM_10_-Busan	2	1.772	1.774	2.155	0.855	0.864	0.853
	5	1.392	2.155	1.519	0.896	0.866	0.896
	7	1.392	1.645	1.649	0.901	0.878	0.869
PM_2.5_-Busan	2	0.951	1.056	0.692	0.896	0.894	0.914
	5	0.609	1.995	0.891	0.934	0.850	0.910
	7	0.607	1.127	1.211	0.940	0.878	0.883
NO_2_-Busan	2	2.606	2.740	2.747	0.617	0.621	0.667
	5	2.205	2.594	2.277	0.667	0.623	0.669
	7	2.140	2.284	2.137	0.680	0.663	0.689

### 3.4. Scatter Plots for the Performance of an Optimal ANN Model with Different Numbers of Nodes in a Hidden Layer 

Figure 4 and Figure 5 illustrate scatter plots with the coefficients of R^2^ between daily mean measured PM_10_, PM_2.5_, and NO_2_ concentrations and ones predicted by the ANN models transferring by a sigmoid activation function from the input layer to the hidden layer and from the hidden layer to the output layer for 2, 5, and 7 neurons in the hidden layer at both Seoul and Busan cities, respectively. 

The linear least-square method is employed to fit the empirical coefficients obtained for all variables by the ANN models. These scatter plots show that regardless of the number of nodes in the hidden layer, the correlation coefficients between the predicted and measured values had almost similar magnitudes. Among them, the highest correlation coefficients between the predicted and measured values of all variables were found in the five nodes of the hidden layer, except for the PM_2.5_ (0.914 in 2 nodes) and NO_2_ (0.689 in 7 nodes) variables in Busan city. Thus, overall, it can be said that the predicted values in the five nodes of the hidden layer are the best validated. 

Especially, correlation coefficients of NO_2_ concentrations in three kinds of hidden nodes show slightly lower values of 0.667 (2 nodes), 0.669 (5 nodes), and 0.689 (7 nodes) than other variables, and widely spreading data distributions from the regression lines. However, the Pearson r regression coefficient exceeded 0.817 (R^2^; 0.667) in the NO_2_ concentrations in both cities, having a maximum value of 0.864 (R^2^; 0.746) in Seoul.

### 3.5. Sensitivity of the Performance of an Optimal ANN Model with Different Numbers of Nodes in a Hidden Layer 

The daily mean observed (measured) and calculated (predicted) values of PM_10_ (μg/m^3^), PM_2.5_ (μg/m^3^), and NO_2_ (ppm ×1000) concentrations estimated by ANN models denote the behavior throughout the one-year period from 1 January to 31 December 2020, as the COVID-19 epidemic year (2020) in Seoul and Busan cities (Figure 6 and Figure 7). The comparisons between the predicted and measured values of PM_10_, PM_2.5_, and NO_2_ concentrations were made using different numbers of hidden neurons-2, 5, and 7 in an ANN model-based a sigmoid activation function. 

In Figure 6, daily variations in PM_10_, PM_2.5_, and NO_2_ concentrations were presented in Seoul city, and their distributions show almost the same patterns and magnitudes in three kinds of nodes, except for in May and June with much smaller predicted values than the observed ones, in part. 

The predicted values of PM_10_ in 2 and 5 nodes of the hidden layer reach nearly zero, which is much deviated from the observed values to some extent, in late April, May, and June, but the predicted values of PM_10_ in 7 nodes well match the observed ones without showing zero value. Similarly, the predicted values of PM_2.5_ (NO_2_) in 5 nodes reach still zero partially in May (May and June), but there are no zero values in the other two nodes. However, in the daily distribution of NO_2_ concentration, the predicted values were overall relatively smaller than the observed ones, regardless of the node number. 

On the other hand, Figure 7 shows the predicted values of PM_10_ and PM_2.5_ concentrations well reflected in their observed values with the almost same patterns and magnitudes in three different kinds of nodes in Busan city in 2020. The predicted values of NO_2_ were very close to the observed values with almost the same decreasing patterns, but with slightly smaller predicted values. As result, through the comparison of the predicted and observed values in Table 4 and Figure 6 and Figure 7, the predicted values are generally well reflected regardless of node numbers in the hidden layer. 

## 4. Conclusions

The prediction of daily averaged PM_10_, PM_2.5_, and NO_2_ Concentrations between two megacities without a lockdown of cities in Korea during the COVID-19 Pandemic Period of 2020 was performed by Artificial neural network models (ANN)-sigmoid activation function, and it gave the following results.

1. Daily and weekly mean concentrations of PM_10_ (PM_2.5_) in 2020 under neither locked down cities, nor limitation of the activities of vehicles and people by the Korean Government has slightly decreased by about 15% (10%) in Seoul city, and 12% (10%) in Busan city, compared to ones in 2019, respectively. NO_2_ concentration has also decreased by about 15% in Seoul, and 12% in Busan, showing a similar significant decline of PM_10_ (PM_2.5_), resulting in more than 10% improvement in the air quality of each city. 

2. The coefficients of determination (R2; (Pearson r)^2^) between the predicted values of daily mean PM_10_, PM_2.5_, and NO_2_ using the ANN models with 2 (5 and 7) nodes in a single hidden layer and their measured values in Seoul were 0.882 (0.896 and 0.892), 0.925 (0.930 and 0.922), and 0.729 (0.746 and 0.734), respectively. 

3. The coefficients of determination in Busan in cases of 2 (5 and 7) hidden nodes were 0.853 (0.896 and 0.869), 0.914 (0.910 and 0.883), and 0.667 (0.669 and 0.689), showing slightly lower coefficients than ones in Seoul.

4. The closeness of the prediction to the observation exceeds 0.932 of Pearson r in both PM_10_ and PM_2.5_, and 0.817 in NO_2_ at two cities. 

5. The artificial neural network models developed by us could perform well in the prediction of dependent variables of PM_10_, PM_2.5_, and NO_2_ in Seoul and Busan cities, even though NO_2_ concentration in Busan city had slightly lower R^2^ between the predicted and observed values than PM_10_ and PM_2.5_ concentrations.

6. As a result, through scatter plots and temporal distributions of the predicted and observed values of PM_10_, PM_2.5_, and NO_2_ concentrations, the predicted values are well reflected in the observed values of each variable, regardless of node numbers in the hidden layer. Overall, the ANN model with 5 hidden nodes was the best to make the predicted values closest to the measured values, and 7 hidden nodes were next followed.

## Data Availability

Daily air quality data without any restrictions after registering personal information through the National Institute of Environmental Research (NIER), the Ministry of Environment of the Korean Government with a website address as https://www.nier.go.kr/NIER/kor/openapi/ (accessed on 10 January 2021).
